# Implication of replicative stress-related stem cell ageing in radiation-induced murine leukaemia

**DOI:** 10.1038/sj.bjc.6605135

**Published:** 2009-06-09

**Authors:** N Ban, M Kai

**Affiliations:** 1 Department of Health Sciences, Oita University of Nursing and Health Sciences, 2944-9 Megusuno, Oita 870-1201, Japan

**Keywords:** replicative stress, stem cell ageing, radiation, acute myeloid leukaemia, C3H/He mice, mathematical model

## Abstract

**Background:**

The essential aetiology of radiation-induced acute myeloid leukaemia (AML) in mice is the downregulation of the transcription factor PU.1. The causative mutation of the PU.1-endocing *Sfpi1* gene consists mostly of C:G to T:A transitions at a CpG site and is likely to be of spontaneous origin. To work out a mechanism underlying the association between radiation exposure and the AML induction, we have hypothesised that replicative stress after irradiation accelerates the ageing of haematopoietic stem cells (HSCs), and the ageing-related decline in DNA repair could affect the spontaneous mutation rates.

**Methods:**

Mathematical model analysis was conducted to examine whether and to what extent the cell kinetics of HSCs can be modified after irradiation. The haematopoietic differentiation process is expressed as a mathematical model and the cell-kinetics parameters were estimated by fitting the simulation result to the assay data.

**Results:**

The analysis revealed that HSCs cycle vigourously for more than a few months after irradiation. The estimated number of cell divisions per surviving HSC in 3 Gy-exposed mice reached as high as ten times that of the unexposed.

**Interpretation:**

The mitotic load after 3 Gy irradiation seems to be heavy enough to accelerate the ageing of HSCs and the hypothesis reasonably explains the leukaemogenic process.

There is substantial evidence that ionising radiation can cause various types of malignancies. Among them, leukaemia is noteworthy because of the high relative risk and the short latency (Preston *et al*, 1994). The mechanism of radiation leukaemogenesis, however, is not yet fully explained despite recent advances in the understanding of genetic alterations in leukaemic cells. It has been speculated that leukaemia-specific gene rearrangements (Schoch and Haferlach, 2002; Mrózek *et al*, 2004) are attributable to the misrepair of initial DNA damage caused by radiation. This notion, however, suffers from the low probability of such events given the random nature of radiation-induced DNA damage. It is also difficult to rationalise the latency period for the disease. Radiation-induced delayed gnomic instability could explain the leukaemogenic process more plausibly, but there is no conclusive evidence of its involvement (Morgan, 2003). The findings of healthy carriers of leukaemia-specific gene rearrangements have lead to a further bold hypothesis that only those carriers are at risk for developing leukaemia when exposed to radiation (Nakamura, 2005).

Radiation-induced acute myeloid leukaemia (AML) in some strains of mice serves as a well-established animal model to explore this issue. The incidence increases with doses up to 3 Gy and the symptoms of the affected mice are similar to those found in human AML (Upton *et al*, 1958; Major, 1979; Mole *et al*, 1983; Resnitzky *et al*, 1985; Seki *et al*, 1991). The leukaemic cells usually carry a deletion of chromosome 2 (Hayata *et al*, 1983; Trakhtenbrot *et al*, 1988; Rithidech *et al*, 1993; Clark *et al*, 1996) and a mutation of the *Sfpi1* gene on the retained homologue (Cook *et al*, 2004; Suraweera *et al*, 2005; Hirouchi *et al*, 2008). These genetic changes result in downregulation of the transcript PU.1, which is crucial for normal myeloid differentiation (Cook *et al*, 2004). Although the *Sfpi1* mutations are often accompanied by additional genetic alterations, such as amplification of *Myc* (Rosenbauer *et al*, 2004; Hirouchi *et al*, 2008), studies with genetically engineered mice show that downregulation or inactivation of PU.1 is sufficient to induce AML (Rosenbauer *et al*, 2004; Metcalf *et al*, 2006). Therefore, radiation is considered to be responsible for either or both of two genetic events, the deletion of chromosome 2 and the mutation of *Sfpi1*.

Several studies have provided circumstantial evidence that radiation can induce AML-type deletions of chromosome 2 (Hayata, 1985; Trakhtenbrot *et al*, 1988; Ban *et al*, 1997; Peng *et al*, 2009), even though it is inconclusive whether the aberrations arise as a consequence of initial DNA damage or through delayed chromosomal instability (Plumb *et al*, 1998; Bouffler *et al*, 2001; Boulton *et al*, 2001). As for the mutation of *Sfpi1*, however, there is no direct data about the involvement of radiation in its induction. The *Sfpi1* mutations found in AMLs are mostly point mutations at codon 235 of the DNA-binding Ets domain. The majority of them are C:G to T:A transitions at a CpG site (Cook *et al*, 2004; Suraweera *et al*, 2005; Hirouchi *et al*, 2008), the most common type of spontaneous mutations (Douglas *et al*, 1994; Ushijima *et al*, 1994). In the case of radiation-induced mutations, large-scale deletions are most frequent (Thacker, 1986; Sankaranarayanan, 1991). Point mutations might predominate among late-arising mutations (Little, 2000), but transversions rather than transitions are expected given that oxidative stress-related genotoxicity directly contributes to those mutations (Wright, 1998; Busuttil *et al*, 2003). For these reasons, the *Sfpi1* mutations of murine AML are likely to be of spontaneous origin.

Normal cells equipped with various forms of repair machinery can replicate and maintain DNA with great accuracy. Nevertheless, DNA is damaged and mutated on repeated replications. This is the case even in haematopoietic stem cells (HSCs) because they accumulate DNA damage after repeated rounds of replications and those mutations are associated with stem cell ageing (Kamminga and de Haan, 2006; Sharpless and DePinho, 2007). With evidence of decline in HSC functional capacity among mice deficient in DNA repair or telomere maintenance, accumulation of DNA damage is postulated to be a cause of stem cell ageing (Rossi *et al*, 2007). At the same time, the mutation accumulation would be accelerated as the cells age because HSCs from old wild-type mice show the decreased expression of DNA repair and maintenance genes (Chambers *et al*, 2007). The aged, damaged stem cells eventually lose repopulating capability and stem cell function, but they continue to proliferate with compromised genomic integrity if they could escape extinction. In this regard, early studies on haematopoietic effects of radiation present intriguing data. The frequency of CFU-S is depressed while the level of cycling is increased over the lifespan of x-irradiated mice (Tejero *et al*, 1988; Lorimore and Wright, 1990). If HSCs also cycle at enhanced rates for a prolonged period of time, the replicative stress will result in premature ageing. The aged stem cells would show a decline in DNA damage repair capacity and are prone to spontaneous mutations.

In this study, we have used a mathematical model analysis to examine whether and to what extent radiation cause replicative stress in HSCs. The differentiation process of haematopoietic cells was expressed in a mathematical model. The cell-kinetics parameters were estimated by fitting the simulation result to the experimental data of CAFC (cobblestone area forming cell) and CFU-G/M (colony forming unit-granulocyte/macrophage) assays. The analysis showed the upregulated cell kinetics for HSCs, which seemed to be sufficient to accelerate ageing of them. Possible leukaemogenic processes have been discussed based on the results, and we have found the most plausible explanation is that the replicative stress-related stem cell ageing plays a role elevating spontaneous mutation rates of HSCs and their progeny.

## Materials and methods

### Model structure

**Figure 1:**
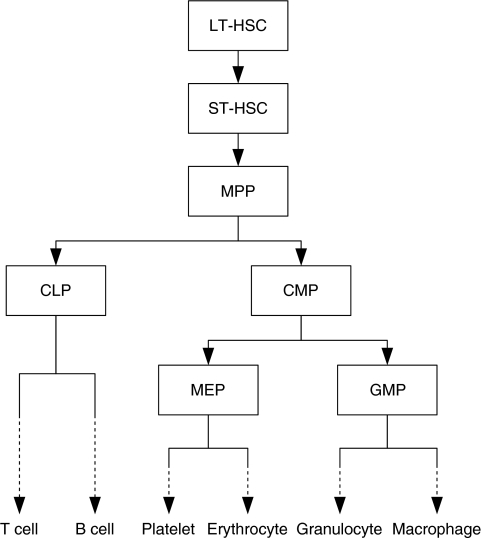
Hierarchy of haematopoietic cells. Long-term reconstituting haematopoietic stem cells (LT-HSCs) are the source of all blood-cell lineages. Subsequent short-term reconstituting stem cells (ST-HSCs) have a limited self-renewal activity and give rise to multipotent progenitors (MPPs). MPPs produce common lymphoid progenitors (CLPs) and common myeloid progenitors (CMPs). CMPs further differentiate into megakaryocyte/erythroid progenitors (MEPs) and granulocyte/macrophage progenitors (GMPs).

Figure 1 illustrates the hierarchy of haematopoietic cells. Although various assay techniques have been developed to measure the frequencies of those cells, the assay data do not necessarily represent the single cell types in the figure. For example, cycling HSCs are not identified as LT-HSC owing to the cell-cycle dependent decline in engraftment ability (Habibian *et al*, 1998; Passegué *et al*, 2005; Bowie *et al*, 2006). There are also transient or fuzzy states between cell types. Applying a linear reservoir model cannot demonstrate massive cell growth through the differentiation process unless unrealistic parameters are assumed. These facts indicate that a simulation with a straightforward modelling of the hierarchy shown in Figure 1 is hard to verify experimentally.

**Figure 2:**
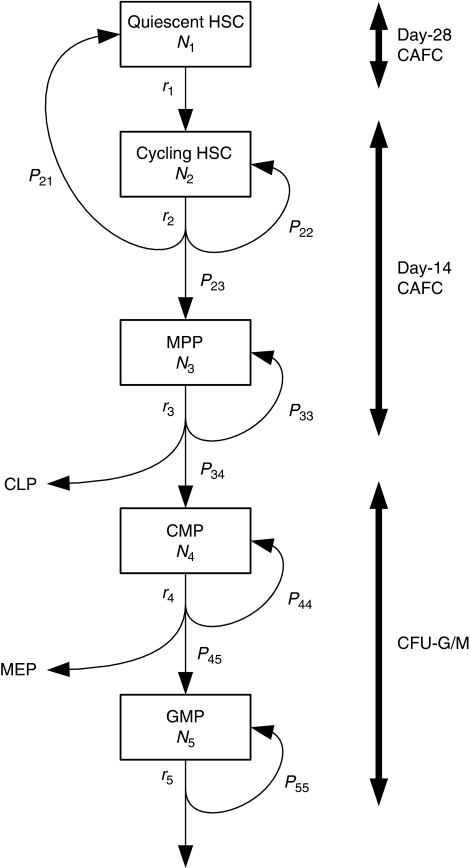
Framework of mathematical model for myelomonocytic haematopoiesis. HSCs that have long-term repopulating ability are mostly in the G_0_ phase and these cells are denoted as quiescent HSCs. Quiescent HSCs recruited into the cell cycle (cycling HSC) replenish themselves or differentiate into MPPs, CMPs, and GMPs in sequence. Equivalent cells in the assays are shown on the right side. *N* denotes the number of cells, *r* the transfer/mitotic rate, and *P* the branching probability.

To overcome these problems without compromising scientific exactitude, we have constructed a mathematical model of myelomonocytic haematopoiesis as shown in Figure 2. In this model, HSCs are divided into quiescent HSCs and cycling HSCs. The former are LT-HSCs in G_0_ phase, which can be quantified by a repopulation assay such as day-28 CAFC. Multipotent progenitor (MPP) in Figure 2 represents ST-HSC and MPP in a limited sense. Both have transient *in vivo* multilineage potential and are thought to share similar cell kinetics. Day-14 CAFC can be an indicator of these cell types as well as cycling HSCs (Ploemacher *et al*, 1991). Common myeloid progenitor (CMP) is equivalent to CFU-GEMM and GMP includes CFU-GM, CFU-G, and CFU-M. The ordinary CFU-G/M assay, therefore, gives a measure of those cells. Each cell compartment has a recurrent flow (self-renewal activity), but only HSCs are presumed to be capable of complete replenishment.

### Mathematical description

In Figure 2, quiescent HSCs recruited into the cell cycle (cycling HSCs) self-renew or differentiate into MPPs. In the case of self-renewal, the HSC returns to the quiescent state or continues to cycle. As self-renewal and differentiation of cells take place on mitoses, these processes are described by ordinary differential equations with respect to time as 
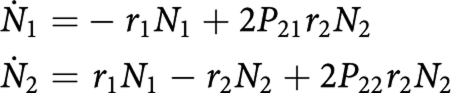
 where *N*_1_ is the number of quiescent HSCs, *N*_2_ the number of cycling HSCs, *r*_1_ the rate of a quiescent HSC transferring to the cell cycle, *r*_2_ the mitotic rate of cycling HSCs, *P*_21_ the probability of a self-renewed HSC returning to the quiescent state, and *P*_22_ the probability of a self-renewed HSC continuing to cycle.

Multipotent progenitor is a progeny of HSC and produces two types of progenitors, common lymphoid progenitor and CMP. The rate of change of MPP frequency is then written as 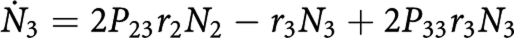 where *N*_3_ is the number of MPPs, *r*_3_ the mitotic rate of MPP, *P*_23_ the probability of an HSC differentiating into MPP, and *P*_33_ the self-renewal probability of MPP (<0.5). Temporal variations of CMPs and GMPs can be described in the same manner.

### Simulation of haematopoiesis

Parameter values used in the simulation are summarised in Table 1. The steady-state condition was assumed in unexposed mice and the values of *r* were estimated from literature data of BrdU incorporation *in vivo* (Bradford *et al*, 1997; Sudo *et al*, 2000; Passegué *et al*, 2005). The branching probability *P* for the self-renewal of each cell type was determined such that the equilibrium stationary state demonstrates the average values of the CAFC assay and the CFU-G/M assay in the unexposed mice. Other *P*-values were calculated by the relational expressions in Table 1 (Spangrude and Johnson, 1990). Details about the parameter values are given in the Supplementary material.

The number of cells in each compartment in the stationary state was calculated by assuming the population size of quiescent HSCs in an adult mouse to be 1 × 10^4^ (Eaves *et al*, 1997). The cell numbers were multiplied by survival fractions to estimate the number of surviving cells just after 3 Gy x-irradiation. Survival fractions for MRA [CFU-S-12], CFU-S-12, CFU-S-7, and CFU-C were calculated by the linear-quadratic model of Meijne *et al* (1991) and were applied to quiescent HSC, cycling HSC/MPP, CMP, and GMP, respectively. The surviving cell numbers were set as the initial values and the time course of the recovery was simulated. Some parameters were modulated within realistic ranges during the recovery. They are the parameters for HSCs, the mitotic rate of MPP, and the self-renewal probability of CMP. The other parameters were unchanged or followed the relational expressions in Table 1.

Finally, the number of cell divisions of each cell type was estimated based on the simulation result. Setting time to be 0 at irradiation, the cumulative number of cell divisions in the *i*th cell compartment until time *t* was calculated by the formula 
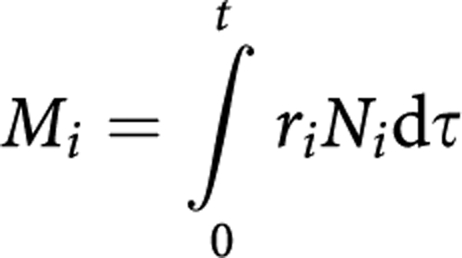
 with the mitotic rate *r*_*i*_ and the number of cells *N*_*i*_ at time *τ*.

All calculations were performed in R using the lsoda function of the odesolve package.

### CAFC and CFU-G/M assays

Male C3H/HeN mice were purchased from Japan Clea and maintained under conventional conditions. At 8–10 weeks of age, they were exposed to 3 Gy of 200 kV X-rays filtered with 0.5 mm Cu and 0.5 mm Al (dose rate 0.47 Gy min^−1^). The irradiated mice as well as age-matched controls were killed at 30, 90, 150, and 300 days post irradiation and bone marrow cells were collected from the femora. Animal experiments were performed with the approval of the Research Ethics Committee of Oita University of Nursing and Health Sciences in conformity with applicable laws and regulations.

For the CAFC assay, bone marrow cells of individual mice were suspended in *α*-MEM (Sigma-Aldrich, Irvine, UK) containing 12.5% foetal calf serum (ThermoTrace, Melbourne, Australia), 12.5% horse serum (ThermoTrace), 10^−5^ M hydrocortisone (Sigma-Aldrich), 100 U ml^−1^ penicillin, and 100 *μ*g ml^−1^ streptomycin (Sigma-Aldrich). The cells were overlaid on MC3T3-G2/PA6 stromal cells (provided by the RIKEN CELL BANK) grown in a collagen-coated 96-well flat-bottom microplate (Nalge Nunc International, Rochester, NY, USA). Six dilution steps of the overlaid cells were made with 3-fold intervals from 1000 to 243 000 cells in 150 *μ*l culture volume, and 16 wells were used for each dilution. Cultures were maintained at 33°C, 5% CO_2_, and fed weekly by a half medium change. On days 14 and 28 of culture, the wells were inspected for cobblestone areas with an inverted microscope TMF (Nikon, Tokyo, Japan). A well was scored as positive if it contained more than one cobblestone area that comprised at least five phase-dark cells. The limdil function of the statmod package for R was used to find the maximum likelihood solutions of the frequencies of the CAFCs.

Part of the bone marrow cells from each mouse was used for the CFU-G/M assay. The cells were diluted with IMDM (Sigma-Aldrich) containing 2% foetal calf serum and were mixed with methylcellulose medium MethoCult GF M3534 (StemCell Technologies, Vancouver, Canada). Two dishes of the semi-solid culture containing 1.5 × 10^4^ bone marrow cells in 1.1 ml were prepared for each mouse and were incubated at 37°C, 5% CO_2_. On day 10 of incubation, the number of colonies containing more than 50 cells was counted on an inverted microscope.

## Results

### Frequencies of primitive haematopoietic cells

**Figure 3:**
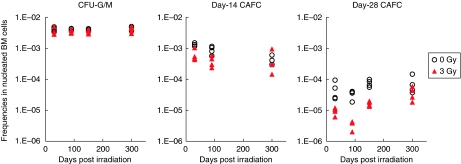
Results of CFU-G/M and CAFC assays. Four mice were used for each experimental condition and the markers represent individual animals. Data are missing for day-14 CAFC of 150 days post irradiation because maximum likelihood solutions were not obtained because of inconsistently varied data (see main text for details).

Figure 3 shows the results of CFU-G/M and CAFC assays. The frequencies of CFU-G/M in the exposed mice are comparable to the unexposed throughout the experimental period, indicating a rapid recovery of the committed progenitors after irradiation. With regard to the CAFC frequencies, data of day-14 cultures for 150 days post irradiation varied so much that the limdil function failed to find maximum likelihood solutions. The day-14 CAFC frequency was also not calculable for a mouse of the control group for 30 days post irradiation because all wells were positive for the cobblestone area. Despite these missing data, Figure 3 clearly shows that the level of primitive CAFCs was suppressed for a few months after exposure. Long-term suppression was remarkable in day-28 CAFCs. The frequency fell down to a tenth of the normal level at 90 days post irradiation and did not fully recover even at 300 days post irradiation.

### Simulation of recovery after irradiation

In the simulation of the recovery process, we first tested for the priority to generate downstream cells rather than self-renewal of HSCs, that is, increasing the branching probabilities for HSCs differentiating into MPPs. This was not effective even in the short term, however, and just exhausted HSCs. Upregulating the transfer/mitotic rates of HSCs and MPPs resulted in quick recovery of CFU-G/M, but the frequency of HSCs was suppressed to a low level at all times if the self-renewal probability of HSC was unchanged. These results show that it is necessary to enhance the self-renewal of HSCs before the committed progenitors are restored. It is also noteworthy that the frequencies of committed cells can vary drastically in response to a subtle change in HSC kinetics, indicating that continuous, fine adjustments are required for the parameters.

**Figure 4:**
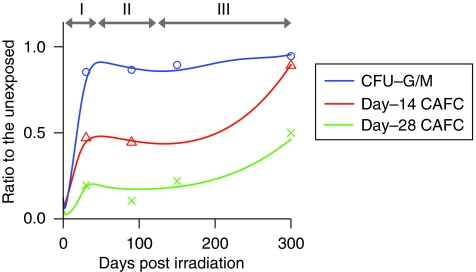
Simulation of temporal changes in CFU-G/M and CAFC frequencies. The lines indicate the simulation results. The markers represent mean values of the experimental data shown in Figure 3 (circle, CFU-G/M; triangle, day-14 CAFC; and cross, day-28 CAFC). ‘Variable’ parameters were continuously modulated with time in the simulation. The time course of the parameter changes can be divided into three prominent phases and are shown above the graph (see main text for details).

Figure 4 displays a simulation result that fits to the assay data. Although the parameters are not determined uniquely, they need to be modulated in a certain way to demonstrate the recovery pattern observed in the experiment. A time course of the parameter changes can be divided into three prominent phases shown in the graph.

Phase I: HSCs and MPPs respond to haematopoietic demands until CFUs-G/M are restored. The probability of HSC self-renewal is increased. Transfer/mitotic rates of HSC and MPP are high.
Phase II: Cell kinetics is downregulated to prevent the overgrowth of CFUs-G/M. The probability of the HSC self-renewal is suppressed. Transfer/mitotic rates are decreased, but still higher than the normal state.
Phase III: HSCs increase gradually whereas the CFU-G/M frequency remains constant. The probability of the HSC self-renewal is moderately increased, but CMP self-renewal is suppressed. Mitotic rates of HSCs and MPPs are gradually increased with time.

### Estimation of the number of cell divisions after irradiation

**Figure 5:**
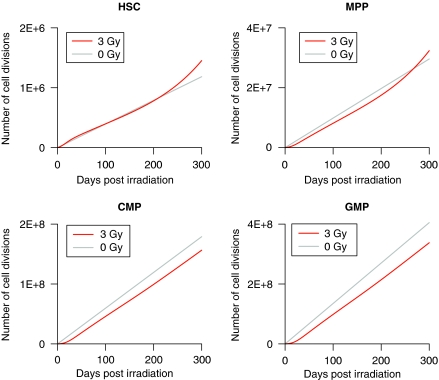
Cumulative number of cell divisions. The number of cell divisions in each cell compartment was calculated using parameter sets that produced the simulation results shown in Figure 4.

**Figure 6:**
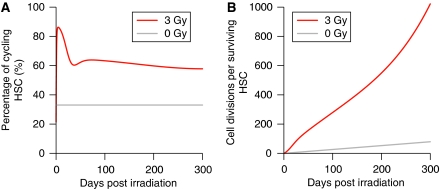
Increased cell cycling in surviving HSCs after irradiation. The percentage of cycling HSCs was calculated from the cell numbers in the quiescent-HSC compartment and the cycling-HSC compartment (**A**). The cumulative number of cell divisions per surviving HSC was calculated by dividing the number of cell divisions of HSCs shown in Figure 5 by the number of HSCs that escaped the cell-killing effect of radiation (**B**).

The number of cell divisions in each cell compartment was calculated in the course of the simulated recovery process. Figure 5 shows the cumulative number of cell divisions with or without irradiation. Although the slope of the curve increases for HSCs and MPPs at phase III of the recovery, there were no striking differences from the control group during 300 days after exposure. However, less than 10% of HSCs survived on exposure to 3 Gy X-rays, meaning that surviving HSCs bear a high mitotic load to achieve a comparable number of cell divisions as a whole. This was showed by the percentage of cycling HSCs, which were elevated just after irradiation and never returned to a normal level (Figure 6A). The number of cell divisions per surviving HSC was remarkably high in that condition, more than 10 times that from unexposed mice (Figure 6B). By 300 days post irradiation, surviving HSCs underwent five times the number of cell divisions that are expected for HSCs from unexposed mice during their entire lifespan, assuming a longevity of 2 years.

## Discussion

We used CAFC assay to get a measure of multipotent haematopoietic cells. The CAFC assay is validated in many applications (e.g., Breems *et al*, 1994; Parmar *et al*, 2005) and is said to be more robust than the widely used CFU assay in some conditions (Olesen *et al*, 2002). Our assay result also demonstrates its robustness showing small inter-individual variations and good agreement in the estimated CAFC frequencies for the unexposed mice with literatures (Ploemacher *et al*, 1989; de Haan *et al*, 1997). Although the missing data points in Figure 3 may reflect a limitation of the culture-based limiting dilution assay, the obtained data are regarded reasonable and reliable. Together with the solid result of the CFU-G/M assay, the experimental data provide a good basis for the mathematical simulation.

In the experiment, day-14 CAFCs and day-28 CAFCs revealed long-term suppression after irradiation, although CFUs-G/M did not show noticeable changes. This is because committed cells are preferentially restored to minimise their influence on circulating blood cells, which are essential for life. The mathematical simulation has showed a quick recovery of CFUs-G/M by vigorously cycling HSCs and MPPs just after irradiation, but the emergent situation settled down as the frequencies of committed cells reached a normal level. Once CFUs-G/M were restored, their frequency remained almost constant and the CAFCs started recovering slowly. It is a contradictory situation where HSCs and MPPs proliferate without affecting the frequencies of the progeny cells. The simulation has found that this can only be achieved by upregulating the kinetics of HSCs/MPPs gradually while suppressing the self-renewal of committed progenitors. The frequency of MPP is suboptimal in that condition even if the cells cycle more rapidly than in the normal state. This indication is consistent with the reported late effects of x-irradiation on CFU-S (Lorimore and Wright, 1990). On the basis of cell kinetics estimated by the simulation, the number of cell divisions of each cell type was calculated. Despite the upregulated cell kinetics for HSCs and MPPs, the cumulative number of cell divisions did not show marked increases. This is because the enhanced proliferation is almost offset by the lower number of cells than in the normal state.

Given the number of cell divisions, it is possible to estimate the spontaneous mutation frequency. Although spontaneous mutations arise from various pathways during normal metabolism, they are fixed through the DNA replication process. If one assumes a constant mutation rate per cell division, mutational events in a cell population follow poisson statistics. Mutation rates in eukaryotic cells are generally ⩽10^−10^ mutations per base pair replicated per generation (Kunkel and Bebenek, 2000). As the *Sfpi1* mutations of AMLs are confined to specific positions of exon 5 (Cook *et al*, 2004; Hirouchi *et al*, 2008), it is reasonable to presume the AML-specific *Sfpi1* mutations occur at the rate of up to 10^−9^ per cell division in normal cells.

**Figure 7:**
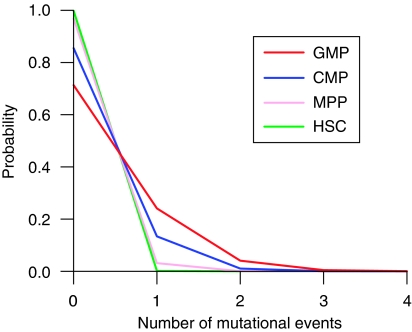
Probability of the AML-specific mutational events in a 3 Gy-exposed mouse during 300 days post irradiation. The probability was calculated from the cumulative number of cell divisions in each cell compartment based on poisson statistics. A constant rate for the AML-specific *Sfpi1* mutation, 1 × 10^−9^ per cell division, was assumed for all cell types.

Figure 7 shows the calculated probability of the AML-specific mutational events in a 3 Gy-exposed mouse for that condition. During the 300 days after exposure, at most one or two mutational events are expected in CMPs and GMPs, but practically zero in HSCs and MPPs. Irradiated C3H/He mice develop AMLs with a median latency of 450–500 days (Seki *et al*, 1991). In conditional gene targeting, AMLs appear 23.5±6.2 weeks after the inactivation of *Sfpi1* (Metcalf *et al*, 2006). Taken together, a rough estimate shows that a malignant cell has to appear by 300 days post irradiation in about a half of leukaemia cases (>10% of the 3 Gy-exposed mice). The calculated probability of the mutational events is too low to explain AML induction in mice, considering that the mutation of *Sfpi1* has to coincide with the deletion of chromosome 2 to form the malignant cell.

There are some possible explanations that reconcile this inconsistency. One is to reconsider the fundamental hypothesis, presuming the mutation of *Sfpi1* is a consequence of radiation exposure. Mutation frequency of a single gene after 3 Gy exposure *in vivo* is estimated to be 10^−5^–10^−4^ based on an extrapolation from high-dose data in *lacZ* transgenic mice (Nakamura *et al*, 2000). Although the value seems to be too small to explain the AML incidence, the frequency could be higher if the relevant chromosomal region has a special chromatin structure and is vulnerable to extrinsic insults. Alternatively, it is possible to suppose that the delayed genomic instability is responsible for the mutagenesis (Morgan, 2003). In either case, the expected mutation spectrum will be different from that found in AML mice as mentioned earlier, but the discrepancy may be attributable to a clonal selection of the mutants. Even so, however, it is unlikely that very limited clones have a survival advantage because the *Sfpi1* mutations required for the AML development are just an inactivation of the gene. Evidence of a total gene deletion or removal of exon 5 developing into the leukaemia (Silver *et al*, 1999; Metcalf *et al*, 2006) implies the particular mutation pattern in the AML mice is not a consequence of clonal selection.

Another possibility is that cells with hemizygous deletion of chromosome 2 acquire a growth advantage and just one or two spontaneous mutagenic events are enough with a large population base of those cells. Haploinsufficiency for *Sfpi1* can bring phenotype alteration enhancing neutrophil progenitor development, but the gene dosage effect is masked *in vivo* by changes in cytokine expression (Dahl *et al*, 2003). In fact, clonal analysis of the chromosomal aberrations in irradiated mice indicates no apparent selective or proliferative advantage of hemizygously deleted cells during 12 months after irradiation (Bouffler *et al*, 1997).

The most plausible explanation will be to allow for the increase in the mutation rate associated with the ageing of stem cells. Our simulation predicts that most of the surviving HSCs are recruited into the cell cycle after 3 Gy exposure, and that the percentage of cycling HSCs remains at ∼60%. This contrasts with the cell kinetics of HSCs in the steady state, in which two-thirds of the population are quiescent. Owing to the increased cycling fraction, together with the enhanced mitotic rate, the surviving HSCs in the exposed mouse undergo the extensive rounds of replication. This will make the cells senescent and the mutation accumulation would be accelerated.

There is considerable evidence that continuous replicative stress accelerates the ageing of stem cells, and that aged stem cells are characterised by compromised genome maintenance. Serial transplantation assays in mice have shown that forced stem cell expansion results in deterioration of HSCs (Harrison *et al*, 1978, 1990; Ross *et al*, 1982; Spangrude *et al*, 1995). Functional changes in deteriorated HSCs are comparable to those observed in aged animals – a reduced repopulating ability and preferential differentiation into myeloid lineages (Sudo *et al*, 2000; Kim *et al*, 2003). Enhanced proliferation of HSCs caused by gene knockouts also leads to a decline of stem cell functions (Cheng *et al*, 2000; Hock *et al*, 2004). Furthermore, a conditional knockout for the *Atr* gene has showed that even homeostatic proliferation can promote stem-cell dysfunction and ageing (Ruzankina *et al*, 2007). Therefore, the excessive number of cell divisions in response to haematopoietic demands after irradiation probably promotes the ageing of surviving HSCs. A recent study by Chambers *et al* has found that the gene expression profile changes as HSCs age, and among the downregulated genes are those involved in the preservation of genomic integrity, such as chromatin remodelling and DNA repair (Chambers *et al*, 2007). A decline in the expression of those genes will manifest as elevated mutation rates.

**Figure 8:**
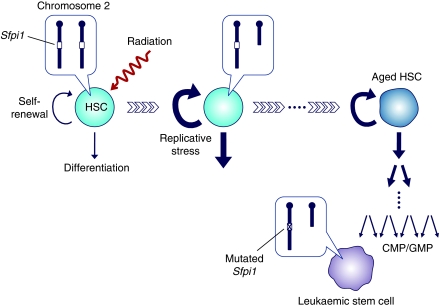
Schematic of proposed model for radiation-induced murine AML. HSCs that escape death by radiation vigourously cycle to reconstitute haematopoiesis. Some of them carry a deletion of chromosome 2, being hemizygous for the *Sfpi1* gene. Enhanced replicative stress promotes ageing of HSCs and the mutation rates gradually increase. On repeated cell divisions with elevated mutation rates, the progeny (CMP or GMP) of an *Sfpi1*-hemizygous HSC spontaneously acquire the AML-specific *Sfpi1* mutation to become a leukaemic stem cell.

We therefore propose a model for murine myeloid leukaemogenesis as shown in Figure 8. Radiation causes cell death of progenitors and stem cells in the haematopoietic system. It also induces chromosomal aberrations and some HSCs survive with a deletion of chromosome 2, being hemizygous for the *Sfpi1* gene. When a substantial fraction of haematopoietic cells are killed by radiation, surviving HSCs vigourously cycle to reconstitute haematopoiesis. Enhanced replicative stress promotes their ageing and mutation rates gradually increase. Random mutations thus accumulate in HSCs during repeated replications unless the mutations bring on a loss of stem cell function. If a leukaemia-specific mutation occurs in an *Sfpi1*-hemizygous cell in due course, the cell becomes a leukaemic stem cell. As the leukaemic stem cells can arise not only from HSCs, but also from CMPs and GMPs (Passegué and Weisman, 2005), those outnumbering cells are more likely to be the origin of AMLs if the elevated mutation rates of HSCs are inherited by the progeny. Although the model does not exclude the possibility that the deletion of chromosome 2 arises from delayed instability, it is more reasonable to presume an early formation of the aberration unless the spontaneous mutation rates increase enormously.

According to this model, the induction pattern of AML could be modified by changing the cell kinetics of haematopoietic cells. For example, the latency of AML development will be longer and the lifetime incidence will decrease if cycling rates of HSCs are slowed down after irradiation. This has actually been showed by dietary restriction in mice. Under caloric restriction, the cycling fraction of haematopoietic stem/progenitor cells is low (Yoshida *et al*, 2006) and senescence of HSCs is postponed (Chen *et al*, 2003). When exposed to radiation, those mice show a delayed onset and lower incidence of AML compared with mice without caloric restriction (Yoshida *et al*, 1997). Moreover, onset delay and significant decrease in incidence are not observed in mice with pre-irradiation restriction, but only with post-irradiation restriction (Yoshida *et al*, 2006). All these findings are consistent with the proposed model.

Finally, we should note the limitations of our mathematical analysis. We consulted the literature for realistic parameter values, but available data were limited and arbitrary values had to be assigned to some parameters. In addition, there are factors not taken into account in the analysis; that is spontaneous cell death/inactivation, cell kinetics change because of natural ageing, strain difference in cell kinetics, and radiation effects on stromal cells. The accumulation of empirical data will enable a more realistic simulation integrating those factors. However, despite these limitations, it is certain that surviving HSCs have to cycle at enhanced rates to recover and maintain haematopoiesis after irradiation. We therefore conclude that the acceleration of HSC ageing because of continuous replicative stress is a major effect of irradiation in murine leukaemogenesis.

## Figures and Tables

**Table 1:** Parameter values used in the simulation

**Parameter**	**Steady state^a^**	**Recovery phase^b^**	**References^c^**
*r*_1_	0.03 [/d]	Variable	Bradford *et al* (1997); Sudo *et al* (2000)
*r*_2_	0.8 [/d]	Variable	Passegué *et al* (2005)
*r*_3_	0.55 [/d]	Variable	Passegué *et al* (2005)
*r*_4_	1.9 [/d]		Passegué *et al* (2005)
*r*_5_	2.5 [/d]		Passegué *et al* (2005)
*P*_21_	0.5−*P*_22_	Variable	
*P*_22_	0.462	Variable	
*P*_23_	1−*P*_21_−*P*_22_		
*P*_33_	0.480		
*P*_34_	0.5 (1−*P*_33_)		
*P*_44_	0.457	Variable	
*P*_45_	0.75 (1−*P*_44_)		Spangrude and Johnson (1990)
*P*_55_	0.320		

a Values for the unexposed steady state.

b Values for 3 Gy-exposed mice. Parameters modulated in response to haematopoietic demands are denoted as ‘variable’.

c References for the values or the relational expressions. For details, see the Supplementary material.
