# Supplementary material for: Implication of replicative stress-related stem cell ageing in radiation-induced murine leukaemia
Source: Br J Cancer. 2009 Jun 9;101(2):363–71. doi: 10.1038/sj.bjc.6605135 (PMC2720201; doi:10.1038/sj.bjc.6605135)
Supplement: Supplementary material [file 6605135x1.doc]

Supplementary material — Details of simulation parameters

**Transfer rate, mitotic rate (*r*)**

The *r* values in the unexposed mice can be estimated from the literature data of BrdU incorporation *in vivo*. Given that there are *N*_0_ cells at time 0, the number of cells that do not mitose by time *t* is *N*_0_ exp(– *rt*). If BrdU is administered to the animal at time 0, the number of BrdU-negative cells at time *t* is approximated by *N*_0_ exp{– *r* (*t* + *τ*)} supposing the total duration of S, G_2_, and M phases to be *τ*. Since *N*_0_ is constant under the steady state condition, the fraction of BrdU-negative cells *F* is described as

$$F=\exp\{-r(t+\tau)\}$$

This can be written to

$r=-\frac{\ln F}{t+\tau}$ equation 1

When *t* >> *τ*

$r=-\frac{\ln F}{t}$ equation 2

Long-term BrdU uptake *in vivo* has been examined for Lin^-^ Rh^lo^ Ho^lo^ (Bradford et al. 1997) and Lin^-^ c-Kit^+^ Sca-1^+^ CD34^-^ HSCs (Sudo et al. 2000). Those HSCs are regarded to be mostly in the G_0_ phase and show very slow BrdU incorporation. Applying equation 2 to that data gives *r*_1_ = 0.03 [/d].

The total HSCs are regarded as the mixture of quiescent cells and cycling cells. Where the fraction of the quiescent (G_0_) cells is denoted as *f*, and the fractions of BrdU-negative cells for quiescent HSCs and cycling HSCs are *F*_1_ and *F*_2_, respectively, *F* for the total HSCs is expressed as

$$F=f F_{1}+(1-f)F_{2}$$

Eventually,

$r_{2}=-\frac{1}{t+\tau}\ln\frac{F-f\exp\{-r_{1}(t+\tau)\}}{1-f}$ equation 3

With the values of *F* and *f* for Lin^-^ c-Kit^+^ Sca-1^+^ Thy1.1^int^ Flk2^-^ cells (Passegué et al. 2005), equation 3 gives *r*_2_ = 0.8 [/d], assuming *τ* to be 8 h. The same literature also provides BrdU incorporation data for MPP, CMP, and GMP. Applying equation 1 and a *τ* of 8 h yields *r*_3_ = 0.55 [/d], *r*_4_ = 1.9 [/d], and *r*_5_ = 2.5 [/d]

**Branching probability (*p*)**

Under the steady state condition,

$$p_{21}+p_{22}=0.5, p_{23}=0.5$$

to keep the number of HSCs constant.

CMPs are more numerous than CLPs in bone marrow, but CLPs can transfer to the lymphatic tissues, and only a part of the CLPs are retained in the marrow. Since information on the transfer rate of CLPs to the lymphatic tissues is not available, the branching probabilities of MPP to CLP and CMP are assumed to be equal for the sake of simplicity. That means

$$p_{34}=\frac{1-p_{33}}{2}$$

The cellular composition is available for Rh^hi^ cells-derived day-8 splenic colonies, 3% erythroid, 47% neutrophil, 13% megakaryocyte, and 37% mixed colonies (Spangrude and Johnson 1990). Based on these data, the branching probability of CMP to GMP is calculated as follows.

$$p_{45}=\frac{47}{3+47+13}\cdot(1-p_{44})=0.75(1-p_{44})$$

**Population size of quiescent HSC**

The value of ~2 × 10^4^ is presented as a stable size of the CRU (competitive repopulating unit) population in the adult mouse (Eaves et al. 1997). It was calculated from a total marrow population of ~2 × 10^8^ cells and CRU frequency of ~1 per 10^4^ cells. CRU is quantified by the repopulating assay *in vivo* and is basically equivalent to day-28 CAFC. In our assay, the frequency of day-28 CAFC was ~5 per 10^5^ cells in unexposed mice. Multiplying this frequency by the size of the marrow population mentioned above, we obtained 1 × 10^4^ as a population size of quiescent HSC.

**Consideration of cloning efficiency**

The cloning efficiency in the HPP-CFC assay is about 30% when Lin^-^ Rh^lo^ Ho^lo^ cells are used as a source (Reddy et al. 1997). The value is 10 - 50% with Thy1^lo^ Lin^-^ Sca-1^+^ cells (Spangrude et al. 1991). However, high replating efficiency (>80%) has been reported for blast colonies (R Tanaka et al. 1995) and the value could be higher in the CFU-G/M assay with quality medium. Appropriate data are not available for the CAFC assay, but there is good agreement in the frequencies between day-28 CAFC and LT-HSC (Ploemacher et al. 1991; Neben et al. 1993). For these reasons, no correction was made for the cloning efficiencies in comparing the simulation result and the assay data.

Bradford GB, Williams B, Rossi R, Bertoncello I (1997) Quiescence, cycling, and turnover in the primitive hematopoietic stem cell compartment. *Exp Hematol* **25**: 445 - 453

Eaves C, Miller C, Cashman J, Conneally E, Petzer A, Zandstra P, Eaves A (1997) Hematopoietic stem cells: inferences from in vivo assays. *Stem Cells* **15 Suppl 1**: 1 - 5

Neben S, Anklesaria P, Greenberger J, Mauch P (1993) Quantitation of murine hematopoietic stem cells in vitro by limiting dilution analysis of cobblestone area formation on a clonal stromal cell line. *Exp Hematol* **21**: 438 - 443

Passegué E, Wagers AJ, Giuriato S, Anderson WC, Weissman IL (2005) Global analysis of proliferation and cell cycle gene expression in the regulation of hematopoietic stem and progenitor cell fates. *J Exp Med* **202**: 1599 - 1611, doi:jem.20050967

Ploemacher RE, van der Sluijs JP, van Beurden CA, Baert MR, Chan PL (1991) Use of limiting-dilution type long-term marrow cultures in frequency analysis of marrow-repopulating and spleen colony-forming hematopoietic stem cells in the mouse. *Blood* **78**: 2527 - 2533

Reddy GP, Tiarks CY, Pang L, Wuu J, Hsieh CC, Quesenberry PJ (1997) Cell cycle analysis and synchronization of pluripotent hematopoietic progenitor stem cells. *Blood* **90**: 2293 - 2299

Spangrude GJ, Johnson GR (1990) Resting and activated subsets of mouse multipotent hematopoietic stem cells. *Proc Natl Acad Sci USA* **87**: 7433 - 7437

Spangrude GJ, Smith L, Uchida N, Ikuta K, Heimfeld S, Friedman J, Weissman IL (1991) Mouse hematopoietic stem cells. *Blood* **78**: 1395 - 1402

Sudo K, Ema H, Morita Y, Nakauchi H (2000) Age-associated characteristics of murine hematopoietic stem cells. *J Exp Med* **192**: 1273 - 1280

Tanaka R, Katayama N, Ohishi K, Mahmud N, Itoh R, Tanaka Y, Komada Y, Minami N, Sakurai M, Shirakawa S (1995) Accelerated cell-cycling of hematopoietic progenitor cells by growth factors. *Blood* **86**: 73 - 79
